# Systematic review of fatigue severity in ME/CFS patients: insights from randomized controlled trials

**DOI:** 10.1186/s12967-024-05349-7

**Published:** 2024-06-03

**Authors:** Jae-Woong Park, Byung-Jin Park, Jin-Seok Lee, Eun-Jung Lee, Yo-Chan Ahn, Chang-Gue Son

**Affiliations:** 1 Korean Medical College of Daejeon University, 62, Daehak‑Ro, Dong‑Gu, Daejeon, 34520 Republic of Korea; 2 Department of Korean Rehabilitation Medicine, College of Korean Medicine, Daejeon University, 176 Daedeok‑Daero, Seo‑Gu, Daejeon, 35235 Republic of Korea; 3 Department of Health Service Management, Daejeon University, Daejeon, Republic of Korea; 4 Research Center for CFS/ME, Daejeon Oriental Hospital of Daejeon University, 176 Daedeok‑Daero, Seo‑Gu, Daejeon, 35235 Republic of Korea; 5 Institute of Bioscience and Integrative Medicine, Daejeon University, 62 Daehak‑Ro, Dong‑Gu, Daejeon, 34520 Republic of Korea

**Keywords:** Myalgic encephalomyelitis (ME), Chronic fatigue syndrome (CFS), Fatigue severity, Meta-analysis

## Abstract

**Background:**

Myalgic Encephalomyelitis/Chronic Fatigue Syndrome (ME/CFS) is a debilitating illness medically unexplained, affecting approximately 1% of the global population. Due to the subjective complaint, assessing the exact severity of fatigue is a clinical challenge, thus, this study aimed to produce comprehensive features of fatigue severity in ME/CFS patients.

**Methods:**

We systematically extracted the data for fatigue levels of participants in randomized controlled trials (RCTs) targeting ME/CFS from PubMed, Cochrane Library, Web of Science, and CINAHL throughout January 31, 2024. We normalized each different measurement to a maximum 100-point scale and performed a meta-analysis to assess fatigue severity by subgroups of age, fatigue domain, intervention, case definition, and assessment tool, respectively.

**Results:**

Among the total of 497 relevant studies, 60 RCTs finally met our eligibility criteria, which included a total of 7088 ME/CFS patients (males 1815, females 4532, and no information 741). The fatigue severity of the whole 7,088 patients was 77.9 (95% CI 74.7–81.0), showing 77.7 (95% CI 74.3–81.0) from 54 RCTs in 6,706 adults and 79.6 (95% CI 69.8–89.3) from 6 RCTs in 382 adolescents. Regarding the domain of fatigue, ‘cognitive’ (74.2, 95% CI 65.4–83.0) and ‘physical’ fatigue (74.3, 95% CI 68.3–80.3) were a little higher than ‘mental’ fatigue (70.1, 95% CI 64.4–75.8). The ME/CFS participants for non-pharmacological intervention (79.1, 95% CI 75.2–83.0) showed a higher fatigue level than those for pharmacological intervention (75.5, 95% CI 70.0–81.0). The fatigue levels of ME/CFS patients varied according to diagnostic criteria and assessment tools adapted in RCTs, likely from 54.2 by ICC (International Consensus Criteria) to 83.6 by Canadian criteria and 54.2 by MFS (Mental Fatigue Scale) to 88.6 by CIS (Checklist Individual Strength), respectively.

**Conclusions:**

This systematic review firstly produced comprehensive features of fatigue severity in patients with ME/CFS. Our data will provide insights for clinicians in diagnosis, therapeutic assessment, and patient management, as well as for researchers in fatigue-related investigations.

**Supplementary Information:**

The online version contains supplementary material available at 10.1186/s12967-024-05349-7.

## Introduction

Myalgic Encephalomyelitis/Chronic Fatigue Syndrome (ME/CFS) is a debilitating illness characterized by core symptoms of chronic fatigue lasting for more than 6 months, unrefreshing sleep, post-exertional malaise (PEM), and cognitive dysfunction [1]. This disorder affects approximately 1% of the global population across all ages, races, and ethnic backgrounds [2]. Also, about 25 to 29% of CFS patients are in a house- or bed-bound state [3], and they have a sixfold higher risk of suicide compared to healthy subjects [4].

Indeed, besides CFS, fatigue is one of the most common morbidities even in the general population, affecting approximately 20% [5]. Additionally, for certain diseases, fatigue is a critical feature of the representative symptoms with high prevalence, for example, 50% in cancer [6], 80% in fibromyalgia [7], and 90% in major depressive disorder (MDD) [8], respectively. Then, CFS has been identified as the most severe form of medically unexplained fatigue and much more severe than other fatigue-associated diseases. Also, CFS patients appeared to have the lowest quality of life (QoL) comparing to subjects suffering from other diseases [9].

On the other hand, fatigue-related medical issues depend on the duration of the fatigue and its severity [10]. Since fatigue is a subjective symptom, the assessment of fatigue severity is a key factor for both patients and physicians [11]. To date, in order to objectify the severity of fatigue among CFS patients, an abundance of questionnaires and assessment tools have been developed, such as the *Chalder Fatigue Questionnaire (CFQ), Multidimensional Fatigue Inventory (MFI),* and *Fatigue Impact Scale (FIS)* [12–14]. Nevertheless, for many healthcare professionals including general practitioners who care for patients with ME/CFS, the difficult process of assessing exact fatigue-related status including, in particular, the severity of fatigue is a clinical challenge due to the lack of standardized global information [15]. Although there have been numerous studies to define the characteristics of CFS, there is no data showing comprehensive features and quantified information on fatigue severity in ME/CFS patients.

Therefore, we aimed to systematically produce the features of fatigue severity and its characteristics using data from randomized controlled trials (RCTs) targeting ME/CFS patients.

## Methods

### Data sources and search terms

In accordance with the Preferred Reporting Items for Systematic Reviews and Meta-Analysis (PRISMA) guidelines [16], a systematic literature survey was performed using four electronic literature databases, PubMed, Cochrane Library, Web of Science, and CINAHL, throughout January 2024. The search keywords was ‘chronic fatigue syndrome’ [MeSH term]. The search terms were “randomised controlled trial” [All Fields] OR “RCT” [All Fields]) AND “chronic fatigue syndrome” [Title] OR “CFS” [Title] in PubMed, while “chronic fatigue syndrome [Record title] AND randomized controlled trial [Title abstract keyword]” in the Cochrane Library. In Web of Science, the search terms were “Randomized Controlled Trial OR RCT [All Fields] and chronic fatigue syndrome OR CFS [All Fields]”, while in CINAHL, it was (“Chronic Fatigue Syndrome” OR “CFS”) AND (“Randomized Controlled Trial” OR “RCT”). The trial type was limited to RCTs, and only the English language was included.

### Eligibility criteria

Articles were screened according to the following inclusion criteria: (1) RCTs or randomized controlled trials, (2) patients with ME/CFS, (3) studies that evaluate the efficacy of ME/CFS intervention, (4) studies that used fatigue-specialized measurements (CFQ; Chalder Fatigue Questionnaire, CIS; Checklist Individual Strength, MFI; Multidimensional Fatigue Inventory-20, FSS; Fatigue Severity Scale, FIS; Fatigue Impact Scale, MFS; Mental Fatigue Scale). The exclusion criteria were as follows: (1) articles with no full text, (2) articles where the number of participants was less than 20, (3) studies without detailed characteristics of patients, (4) studies that did not use fatigue-specialized measurements (CFQ, CIS, FSS, FIS, MFI, MFS).

### Review process and data extraction

The authors conducted a search of databases to identify potentially eligible studies. Subsequently, the full texts of potentially eligible studies were independently screened and crosschecked by the authors. In line with our study's objective to analyze fatigue severity in ME/CFS patients, we included data from all participants in both the intervention and control groups at the initial enrollment time point of each RCT. We extracted the following data from each study: number of ME/CFS patients, sex information, age, ME/CFS diagnostic case definitions, fatigue assessment tools, mean and standard deviations of baseline fatigue scores derived from each assessment tool and 3 different types of fatigue domain (physical fatigue, mental fatigue, and cognitive fatigue). Also we noted details regarding treatment type and duration, publication year, and country where each study was conducted.

### Assessment of study quality, heterogeneity and publication bias

To evaluate the study quality, we used the Cochrane Risk of Bias tool 2 (RoB2), which examines five key areas: the randomization process, deviations from planned interventions, missing outcome data, outcome measurement, and the selection of reported results [17]. The results are reported in Additional file 2: Fig. S2. In cases of high risk of bias, a sensitivity analysis was applied (Additional file 6: Table S3). This analysis removed high-risk bias studies to affirm the stability of our meta-analysis findings, in line with the guidelines provided by the Cochrane RoB2.

For assessing heterogeneity between studies, we employed the I^2^ statistic to evaluate variability among each item. Subsequently, we chose the DerSimonian and Laird method to implement a random-effects model when heterogeneity within each item exceeded 50%, and a fixed-effects model for data showing less than 50% heterogeneity [18]. This model was chosen for its capability to account for both within-study and between-study variability in the overall analysis. Publication and reporting bias potential was evaluated through the utilization of funnel plots and Egger’s test. The results are shown in Additional file 1: Fig. S1 and Additional file 5: Table S2 [19]. Also, we used the PRISMA checklist to assist reviewers in understanding how the review was conducted (Additional file 8).

### Meta-analysis for assessment of fatigue severity in ME/CFS patients

For easy and intuitive presentation of fatigue severity, baseline fatigue scores of ME/CFS patients were converted into a scale of 0 to 100 points based on the characteristics of each assessment tool (Additional file 4: Table S1). To obtain converted scores, we first computed the mean and standard deviations of the raw scores for each treatment and control group. We then normalized these means to a scale of minimum 0 to maximum 100 points, aligning them with the scoring of each assessment tool. We used only baseline fatigue scores of the treatment and control groups because our study focused on investigating the fatigue features of ME/CFS patients rather than assessing the effectiveness of treatment. After this process, we conducted a meta-analysis, calculating and analyzing the mean and 95% confidence interval (CI) of baseline fatigue scores of ME/CFS patients by age, fatigue domain, intervention type, case definitions, weighting them based on sample size (Table 2). We constructed a linear mixed effect model for understanding the correlation between fatigue severity and others factors such as age, continent, assessment tool, case definition and intervention (Fig. 2C, D and Additional file 7: Table S4). To assess the robustness of synthesized results, we also conducted an additional meta-analysis according to the results of quality assessment and publication bias. The results are in Additional file 5: Table S2 and Additional file 6: Table S3. All statistical analyses were performed using the “meta” package (by Guido Schwarzer) in R version 4.2.1. All analyses applied p < 0.05 for statistical significance.

## Results

### General characteristics of the selected RCTs

**Fig. 1:**
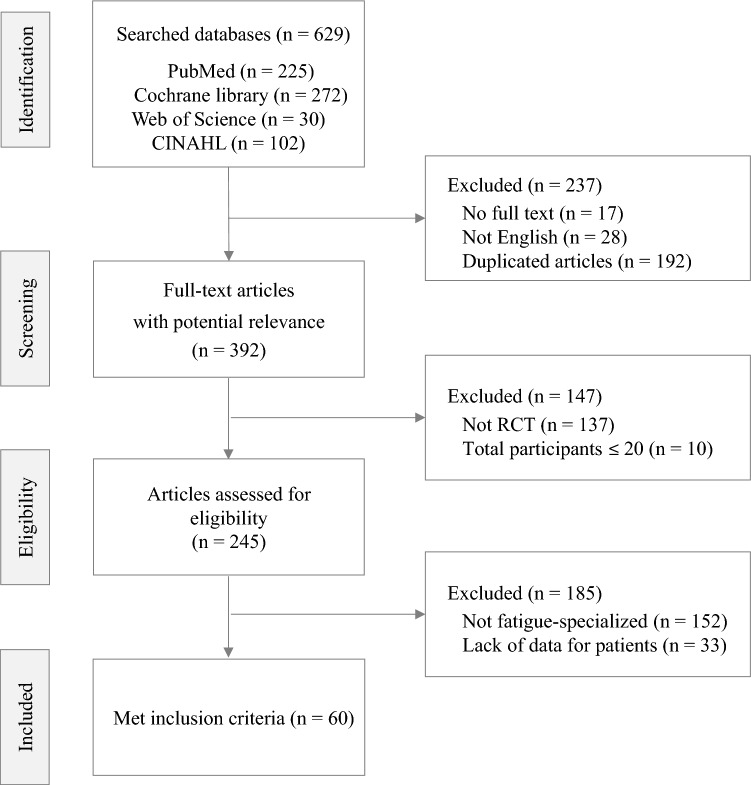
Flow chart of study selection diagram. n: Number of study

A total of 629 articles were firstly identified from 4 database (PubMed, Cochrane databases, Web of Science, and CINAHL) and 60 articles met the inclusion criteria of this review (Fig. 1). In 60 RCTs, 7088 patients with ME/CFS (male 1815, female 4532 and no information: 741, mean age: 37.0 ± 10.1) participated, which were consisted of 54 RCTs for adult patients (n = 6706) and 6 RCTs for minor subjects (n = 382). Twenty-one RCTs evaluated the efficacy of pharmacological interventions, while 39 RCTs were conducted to evaluate non-pharmacological interventions (Table 1). The mean treatment period was 37.0 ± 10.1 weeks (data not shown). All studies were published between 2001 and January 2024 and were conducted across 15 countries. Upon a quality assessment, 45 studies (75%) were classified as having a low risk or some concerns (Additional file 2: Fig. S2).

**Table 1:** Summary of study characteristics

Item	N. of studies	N. of participants (mean ± SD)			
		Total	Male	Female	No information
Total study	60	7088 (118 ± 101)	1815 (34 ± 42)	4532 (85 ± 68)	741 (105 ± 98)
Age					
Adults (39 ± 7.4)	54	6706 (124 ± 104)	1711 (36 ± 44)	4254 (90 ± 71)	741 (105 ± 98)
Minors (15 ± 0.9)	6	382 (63 ± 16)	104 (17 ± 12)	278 (46 ± 13)	0
Type of fatigue					
Physical	16	1207 (75 ± 40)	256 (19 ± 14)	784 (60 ± 36)	167 (55 ± 30)
Mental	16	1207 (75 ± 40)	256 (19 ± 14)	784 (60 ± 36)	167 (55 ± 30)
Cognitive	8	534 (66 ± 35)	60 (12 ± 9)	307 (61 ± 47)	167 (55 ± 30)
Type of intervention					
Pharmacology	21	1382 (65 ± 27)	266 (15 ± 9)	898 (52 ± 29)	218 (54 ± 24)
Psychiatric drugs	4	235 (58 ± 3)	53 (13 ± 5)	182 (45 ± 8)	0
Mitochondria modulators	5	372 (74 ± 42)	6 (3 ± 4)	169 (84 ± 84)	197 (65 ± 12)
Nutrients	3	161 (53 ± 3)	58 (19 ± 3)	103 (34 ± 4)	51 (51 ± 0)
Antiviral drugs	2	94 (47 ± 24)	36 (18 ± 11)	58 (29 ± 12)	0
Others	7	520 (74 ± 27)	113 (18 ± 13)	386 (64 ± 17)	21
Non-Pharmacology^a^	39	5706 (146 ± 114)	1549 (43 ± 48)	3634 (100 ± 76)	523 (174 ± 125)
CBT^b^	19	3236 (170 ± 138)	881 (48 ± 52)	2123 (117 ± 96)	232
GET^c^	4	1078 (269 ± 143)	186 (62 ± 44)	660 (220 ± 128)	232
Self-care	6	761 (126 ± 85)	85 (17 ± 14)	415 (83 ± 48)	261
TKM/TCM^d^	7	1074 (153 ± 95)	452 (64 ± 63)	622 (88 ± 38)	0
Others	5	268 (53 ± 29)	58 (14 ± 18)	180 (45 ± 16)	30
Case definition					
1994 CDC^e^	56	6409 (114 ± 91)	1630 (33 ± 42)	4038 (82 ± 57)	741 (105 ± 98)
Oxford	8	1230 (153 ± 151)	246 (35 ± 37)	752 (107 ± 123)	232
Canadain criteria	2	367 (183 ± 65)	57 (28 ± 17)	310 (155 ± 48)	0
ICC^f^	1	62 (62 ± 0)	10 (10 ± 0)	52 (52 ± 0)	0
Not defined	2	97 (48 ± 6)	31 (15 ± 6)	66 (33 ± 0)	0
Assessment tool^g^					
CFQ	20	2467 (123 ± 110)	513 (27 ± 25)	1722 (90 ± 86)	232
CIS	20	2817 (140 ± 112)	769 (40 ± 49)	1787 (94 ± 65)	261
FIS	6	430 (71 ± 38)	29 (9 ± 11)	204 (68 ± 65)	197 (12 ± 65)
FSS	5	917 (183 ± 88)	393 (78 ± 70)	524 (104 ± 26)	0
MFI	8	395 (49 ± 26)	101 (16 ± 12)	243 (40 ± 16)	51 (25 ± 6)
MFS	1	62 (62 ± 0)	10 (10 ± 0)	52 (52 ± 0)	0
Continent					
Europe	45	5500 (122 ± 107)	1225 (32 ± 39)	3534 (93 ± 76)	741 (105 ± 98)
Asia	12	1406 (117 ± 86)	547 (45 ± 52)	859 (71 ± 38)	0
North America	2	90 (45 ± 21)	18 (9 ± 1)	72 (36 ± 22)	0
Africa	1	92 (92 ± 0)	25 (25 ± 0)	67 (67 ± 0)	0
Publication year					
Before 2010 (~ 2009)	11	1062 (96 ± 66)	375 (34 ± 32)	687 (62 ± 38)	0
Since 2010 (2010 ~)	49	6026 (122 ± 107)	1440 (34 ± 44)	3845 (91 ± 73)	741 (105 ± 98)

*N Number

^a^ 2 out of the 39 non-pharmacological studies applied both CBT and GET interventions simultaneously. We calculated them separately for each item below

^b^*CBT* Cognitive Behavior Therapy

^c^*GET* Graded Exercise Therapy

^d^*TKM/TCM* Traditional Korean Medicine/Traditional Chinese Medicine

^e^*CDC* Centers for Disease Control and Prevention

^f^*ICC* International Consensus Criteria

^g^*CFQ* Chalder Fatigue Questionnaire, *CIS* Checklist Individual Strength, *FIS* Fatigue Impact Scale, *FSS* Fatigue Severity Scale, *MFI* Multidimensional Fatigue Inventory, *MFS* Mental Fatigue Scale

### Overall fatigue severity in participants with ME/CFS

**Fig. 2:**
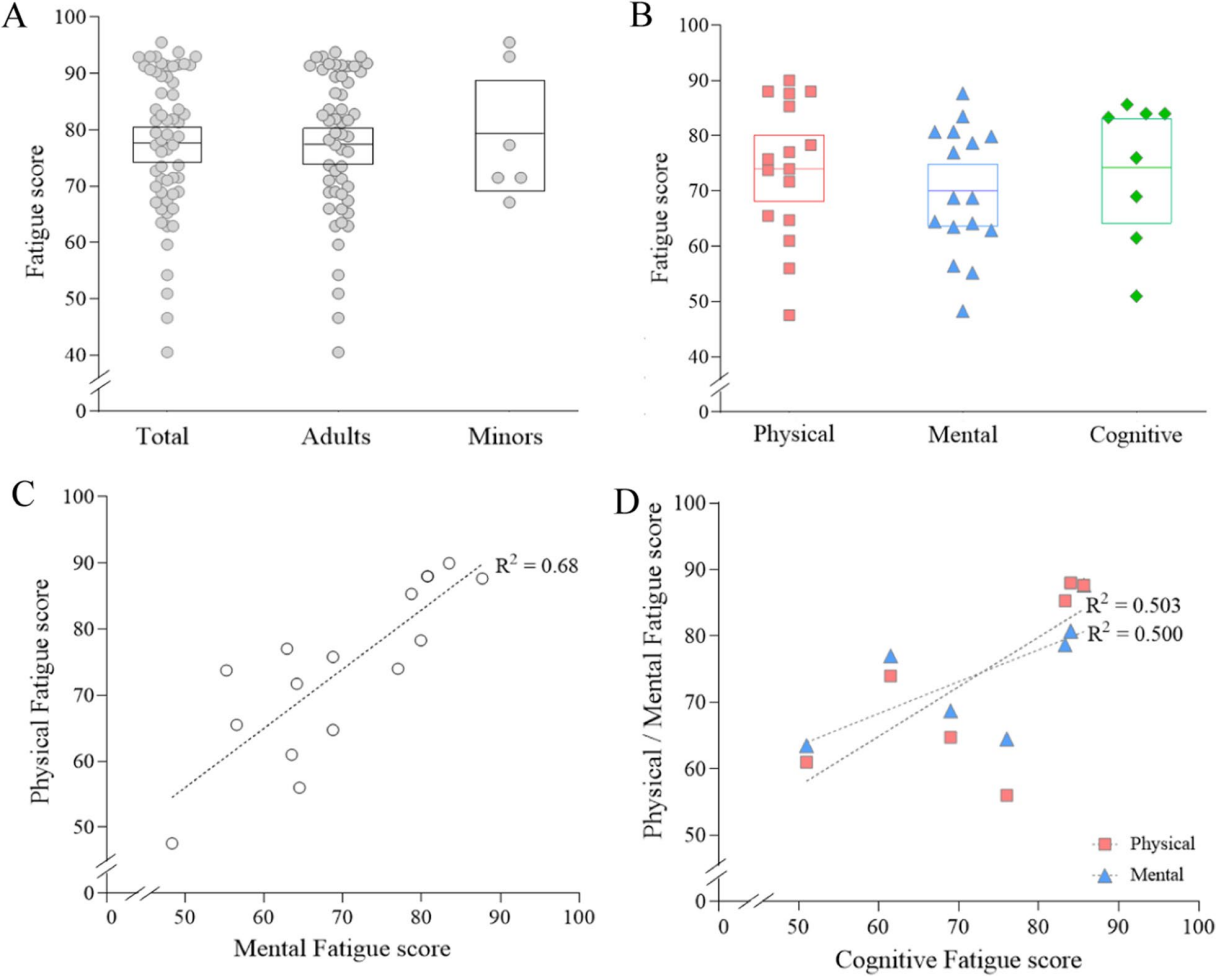
Fatigue severity by age and domain of fatigue. Fatigue severity (out of 100) was calculated and analyzed by subgroups of age (**A**) and fatigue domains (**B**). The correlation among domains of fatigue was shown in (**C**) and (**D**). Each dot represents the value of each study included in this article. The mean score was represented by a horizontal line inside the square, while the 95% CI was depicted by the range of square. *Meta-analysis was done together for each subgroup

Among the 60 RCTs, the overall fatigue severity in the total 7,088 participants with ME/CFS was 77.9 (95% CI 74.7–81.0). The fatigue severity in adult patients (6706 participants from 54 RCTs) was 77.7 (95% CI 74.3–81.0), compared to 79.6 (95% CI 69.8–89.3) in 382 adolescent patients from 6 RCTS, respectively (Fig. 2A; Table 2). The result of forest plot by age was shown in Additional file 3: Fig. S3A. Unfortunately, no RCT presented fatigue severity-related data separately for each male and female.

**Table 2:** Mean and 95% CI of fatigue score in ME/CFS patients

Item	Fatigue score	
	Mean [95% CI]	I^2^
Total study	77.9 [74.7; 81.0]	99.7%
Age		
Adults (40 ± 5.7)	77.7 [74.3, 81.0]	99.7%
Minors (15 ± 0.9)	79.6 [69.8, 89.3]	98.2%
Type of fatigue		
Physical	74.3 [68.3, 80.3]	98.4%
Mental	70.1 [64.4, 75.8]	98.4%
Cognitive	74.2 [65.4, 83.0]	98.4%
Type of intervention		
Pharmacology	75.5 [70.0, 81.0]	99.0%
Psychiatric drugs	72.1 [57.6, 86.6]	99.1%
Mitochondria modulators	76.6 [64.3, 89.0]	96.9%
Nutrients	85.5 [79.1, 92.0]	93.8%
Antiviral drugs	77.3 [74.1, 80.6]	14.0%
Others	71.4 [60.2, 82.7]	99.4%
Non-pharmacology	79.1 [75.2, 83.0]	99.7%
CBT^a^	83.8 [79.4, 88.3]	99.7%
GET^b^	77.2 [68.5, 85.8]	99.7%
Self-care	85.7 [75.6, 95.8]	96.6%
TKM/TCM^c^	64.3 [58.2, 70.3]	95.6%
Others	74.1 [64.0, 84.2]	97.7%
Case definition		
1994 CDC^d^	77.8 [74.5, 81.1]	99.7%
Oxford	77.1 [71.0, 83.1]	99.5%
Canadain criteria	83.6 [69.7, 97.6]	99.7%
ICC^e^	54.2 [51.3, 57.2]	0
Not defined	68.3 [63.2, 73.5]	83.8%
Assessment tool^f^		
CFQ	73.2 [70.0, 76.4]	98.8%
CIS	88.6 [85.4, 91.8]	97.7%
FSS	70.8 [60.6, 80.9]	99.7%
FIS	78.9 [71.6, 86.3]	93.7%
MFI	68.8 [56.4, 81.1]	98.9%
MFS	54.2 [51.3, 57.2]	0
Continent		
Europe	80.3 [76.9, 83.7]	99.4%
Asia	66.6 [61.1, 72.1]	96.9%
North America	83.2 [76.4, 90.1]	79.0%
Africa	95.5 [94.0, 97.0]	0
Year		
Before 2010 (~ 2009)	84.8 [79.1, 90.5]	98.2%
After 2010 (2010 ~)	76.3 [72.7, 79.8]	99.6%

*N* number

^a^*CBT* Cognitive Behavior Therapy

^b^*GET* Graded Exercise Therapy

^c^*TKM/TCM* Traditional Korean Medicine/Traditional Chinese Medicine

^d^*CDC* Centers for Disease Control and Prevention

^e^*ICC* International Consensus Criteria

^f^*CFQ* Chalder Fatigue Questionnaire, *CIS* Checklist Individual Strength, *FIS* Fatigue Impact Scale, *FSS* Fatigue Severity Scale, *MFI* Multidimensional Fatigue Inventory, *MFS* Mental Fatigue Scale

### Fatigue severity in participants with ME/CFS by domain of fatigue

When we recalculated fatigue severity as maximum of 100 points (indicating an unendurable level) according to three domains of fatigue (physical, mental, cognitive fatigue), the ‘mental’ fatigue was the lowest (70.1, 95% CI 64.4–75.8), compared to ‘physical’ fatigue (74.3, 95% CI 68.3–80.3) and ‘cognitive’ fatigue (74.2, 95% CI 65.4–83.0), respectively (Fig. 2B; Table 2). These three domain-related fatigue levels were well correlated, such as R^2^ = 0.68 (p < 0.0001) between physical and mental fatigue (Fig. 2C and D). The result of forest plot by fatigue domain was represented in Additional file 3: Fig. S3B.

### Fatigue severity in participants with ME/CFS by intervention type

**Fig. 3:**
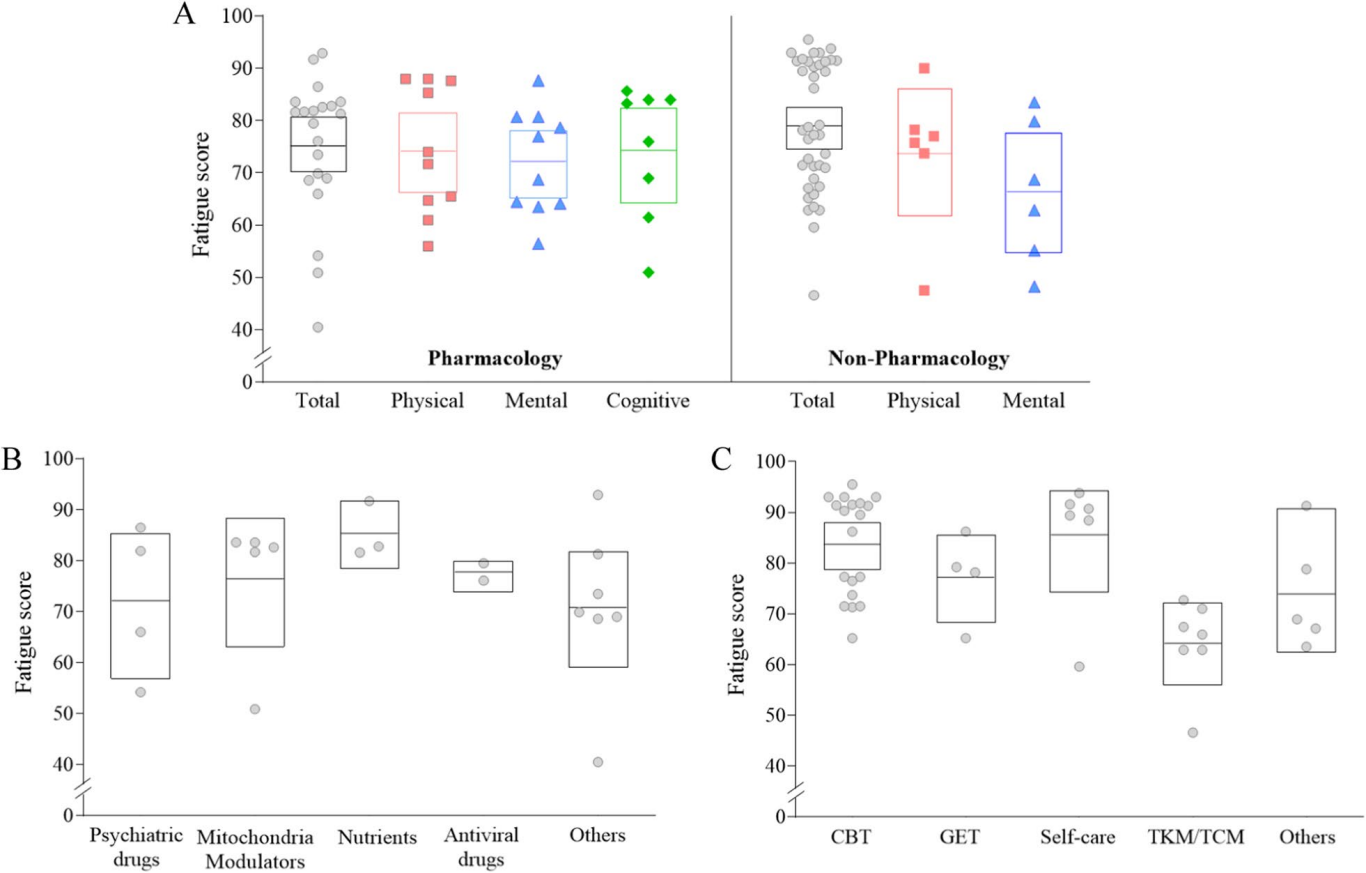
Fatigue severity by intervention type. Fatigue severity (out of 100) was calculated and analyzed by intervention type (A). The results by subgroups of pharmacological and non-pharmacological research are presented in (B) and (C), respectively. Each dot indicates the value of each study included in this article. The mean score was represented by a horizontal line inside the square, while the 95% CI was depicted by the range of square. *Meta-analysis was done together for each subgroup

When we analyzed fatigue severity of ME/CFS patients by intervention type, the total fatigue severity in participants for the non-pharmacological intervention (39 RCTs) was slightly higher (79.1, 95% CI 75.2–83.0) than those for the pharmacological intervention (75.5, 95% CI 70.0–81.0 from 21 RCTs). As we expected, the overall feature of the fatigue domain-related scores were similar with data from total the 60 RCTs, showing lower score in ‘mental fatigue’ than ‘physical’ or ‘cognitive fatigue’ in both pharmacological and non-pharmacological intervention RCTs, respectively (Fig. 3A).

Regarding fatigue severity in ME/CFS patients according to kinds of pharmacological interventions, patients enrolled in nutrients-derived RCTs had the highest fatigue severity (85.5, 95% CI 79.1–92.0) followed by mitochondria modulators (76.6 95% CI 64.3–89.0), antiviral drugs (77.3, 95% CI 74.1–80.6), and psychiatric drugs (72.1, 95% CI 57.6–86.6) (Fig. 3B; Table 2). Meanwhile, patients enrolled in RCTs of ‘self-care’ (85.7, 95% CI 75.6–95.8) had the highest fatigue severity, followed by cognitive behavior therapy (CBT) (83.8, 95% CI 79.4–88.3), and graded exercise therapy (GET) (77.2, 95% CI 68.5–85.8), among RCTs with non-pharmacological interventions (Fig. 3C; Table 2). The result of forest plot by intervention type was shown in Additional file 3: Fig. S3C and Fig. S3D. In linear-mixed effect model,

### Fatigue severity in participants with ME/CFS by case definition

**Fig. 4:**
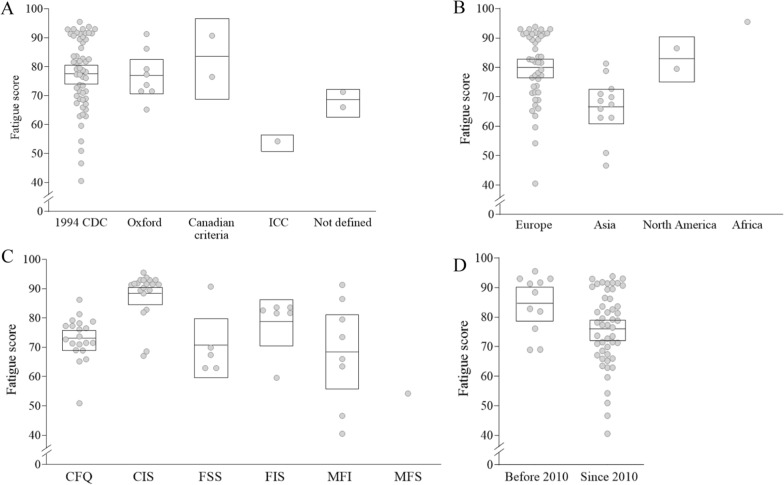
Fatigue severity by case definition of ME/CFS, continent, fatigue assessment tool and publication year. Fatigue severity (out of 100) was calculated and analyzed by case definition of ME/CFS (**A**), continent where the patients lived (**B**), fatigue assessment tool (**C**), and publication year (**D**). Each dot represents the value of each study included in this article. The mean score was represented by a horizontal line inside the square, while the 95% CI was depicted by the range of square. *Meta-analysis was done together for each subgroup

From the analyses for fatigue severity of ME/CFS patients by case definition, no notable difference was observed between two most adapted tools (Table 1), 1994 CDC criteria (77.8, 95% CI 74.5–81.1, 55 RCTs) and Oxford criteria (77.1, 95% CI 71.0–83.1, 8 RCTs). In particular, patients enrolled by Canadian criteria exhibited the highest fatigue severity (83.6, 95% CI 69.7–97.6, 2 RCTs), and conversely, the lowest score was observed in patients by International Consensus Criteria (ICC) (54.2, 1 RCT) (Fig. 4A; Table 2).

### Fatigue severity in participants with ME/CFS by continents

Regarding the countries where studies were conducted, the highest fatigue severity was observed in RCTs conducted in Africa (95.5, but only one RCT) and in Europe (80.3, 95% CI 76.9–83.7, 45 RCTs), compared to the relatively lower scores in Asia (66.6, 95% CI 61.1–72.1) and North America (83.2, 95% CI 76.4–90.1) (Fig. 4B; Table 2).

### Fatigue severity in participants with ME/CFS by assessment tool

Fatigue severity in ME/CFS patients varied according to 6 fatigue assessment tools. Both CIS and CFQ were most frequently adapted in equally 20 RCTs (Table 1), and then fatigue scores were highest in patients assessed by CIS (88.6, 95% CI 85.4–91.8), but 73.2 (95% CI 70.0–76.4) in CFQ. The lowest fatigue sore was observed in patients assessed by MFS (54.2, only one RCT) and MFI (68.8, 95% CI 56.4–81.1), respectively (Fig. 4C; Table 2).

## Fatigue severity in participants with ME/CFS by publication year

When we compared the fatigue severity by publication year, patients participating in 11 RCTS before 2010 presented a more severe fatigue score (84.8, 95% CI 79.1–90.5), compared to 49 RCTs thereafter (76.3, 95% CI 72.7–79.8) (Fig. 4D; Table 2).

## Discussion

In general, fatigue is the most common comorbidity of various diseases and disorders, which impairs the quality of life in diseased individuals. Fatigue sometimes plays a risky factor in the progression of diseases, such as cancer [20], fibromyalgia [7], and major depressive disorder (MDD) [8]. However, for the patients suffering from ME/CFS, fatigue itself is the inherent condition, as the most debilitating illness [21]. Due to this reason, the accurate assessment of fatigue severity is a critical issue in the diagnosis and management of ME/CFS patients in clinical fields and the process of clinical trials [11]. Nevertheless, there is no comprehensive knowledge about the global features of fatigue severity in ME/CFS patients yet.

In order to systematically investigate the features of fatigue severity, we analyzed fatigue severity-related data from RCTs in which ME/CFS patients participated globally. Due to the subjective nature of fatigue symptoms, severity assessment usually relies on patients-reported questionnaires [12]. Among the 60 RCTs finally selected, 6 types of assessment tools were adopted, such as CFQ, CIS, and MFI (Table 1). Each tool has the unique characteristics in terms of questionaries and scoring scales, likely CFQ consisting of 11 questionnaires giving a maximum score of 33 points [13] and MFI consisting of 20 questionnaires giving a maximum score of 100 points [22]. In this study, for easy and intuitive presentation of fatigue severity, we converted the baseline fatigue scores derived from different tools in each RCT to a maximum score of 100 points and conducted meta-analysis. From the data of 60 studies involving 7,088 ME/CFS patients, the overall fatigue level of total patients was 77.9 (95% C I 74.7–81.0) (Fig. 2A).

Regarding the clinical relevance of the 77.9 point fatigue score, one study presented the impact on daily life performance compared to an MFI-derived average fatigue score of 73.8 ± 13.6 for 150 ME/CFS patients [23]. These patients exhibited reduced activity by 50% in 92% of them and were unable to maintain full-time work or attend school in 82%. Moreover, almost half of them (48%) were bedridden or unable to participate in any productive tasks during the period of peaked fatigue, respectively. These findings are very comparable to the known features of the impaired life activity in ME/CFS patients, with approximately 27% of individuals with severe ME/CFS being bedridden, and 57% experiencing either housebound or bedridden status for more than six years [3, 24]. Also, 21.9% of patients with ME/CFS are working part-time jobs [25] and 53.4% of them are unemployed [26]. The medical impact of fatigue severity would vary depending on the types of diseases. For example, a study reported that fatigue scores over 60 points, assessed using the MFI in patients with Parkinson’s disease were considered to have clinically severe fatigue [27]. Also, in another study, the fatigue severity of Critical Illness Polyneuropathy (CIP) patients who were transferred from acute care Intensive Care Unit (ICU) to post-acute ICU was 55.9, assessed by MFI [28]. When comparing fatigue severity among patients with other diseases in RCT data, fatigue score was 73.4 in those with fibromyalgia [29], 50.5 in MDD [30], by MFI-assessed score. It is well acknowledged that fibromyalgia leads to severe fatigue and even has overlapping characteristics with ME/CFS [31], while depression, fatigue and/or pain are commonly accompanied within individuals suffering from MDD [32, 33].

Fatigue is acknowledged as subjective, variable, and influenced by multiple factors, focusing on the unpleasant, distressing, and persistent feeling of tiredness, weakness, or exhaustion experienced [34]. Because of the complexity of fatigue, it is necessary to classify fatigue into various dimensions such as physical, mental, and cognitive to understand it comprehensively. When we performed the sub-analyses, the three different domains of fatigue showed the very similar score with total fatigue, likely 74.3 in physical, 70.1 in mental, and 74.2 in cognitive fatigue score, respectively (Fig. 2B). Also, these three domain-related fatigue levels showed a highly positive correlation (Fig. 2C, D). According to previous research, the primary symptom of fatigue of ME/CFS impacts both physical and cognitive activities, often leading to an extended exacerbation following activities [35]. In fact, PEM, a core symptom of ME/CFS, is raised by any of physical, mental or cognitive activity [36]. Also, the majority of ME/CFS patients experience not only limitations for doing daily activities but also emotional exhaustion and prolonged cognitive activities simultaneously [37]. These results indicate that each fatigue domain cannot be interpreted as separate options but as a systemic phenomenon [38]. There is also research emphasizing that overall impairment of well-being in ME/CFS patients was related to diverse types of fatigue [39].

Fatigue prevalence and severity could be affected by gender, age, ethnicity, and cultural backgrounds [40, 41]. In general, adults tend to exhibit higher levels of fatigue than adolescent patients due to the elderly’s underlying pathogenic conditions and psychological aspects of aging [42, 43]. However, our data shows a 1.9-point higher fatigue score in adolescents compared to adults (Fig. 2A; Table 2). This discrepancy might be attributed to the relatively fewer studies for adolescents (6 RCTs) and the enrollment of individuals with particularly significant levels of fatigue, as noted by the authors [44]. Regarding gender difference, it is well acknowledged that females are predominant and more sensitive to fatigue, with approximately 1.5-fold higher prevalence not only in the general population [5] but also in patients with ME/CFS [45], along with higher fatigue levels in females. Only 4 RCTs exclusively targeted female patients (4 RCTs, n = 326), which presented higher scores of fatigue severity 84.4 (95% CI 76.2–96.2) compared to 77.4 (95% CI 74.1–80.6) from the rest 56 RCTs (data not shown). When comparing fatigue severity by ethnicity, patients from European countries showed higher fatigue severity (80.3) than those from Asian countries (66.6), respectively (Fig. 4B; Table 2). Unfortunately, our present study could not provide additional characteristics related to ethnicity or socioeconomic status due to absence of data from RCTs.

Because our study is based on the RCTs, we attempted to compare the fatigue severity of ME/CFS patients according to intervention. Fatigue severity was slightly higher in participants in non-pharmacological studies (39 RCTs, 79.1) than in those of pharmacological studies (21 RCTs, 75.5) (Fig. 3A; Table 2). Due to the absence of proven therapeutics for ME/CFS, various trials have been performed since the first RCT using GET [46]. Our previous systematic review demonstrated the predominance of pharmacological RCTs in the 1990s and 2000s comparing to non-pharmacological interventions thereafter [47]. In present results, we could find that fatigue severity in participants undergoing ‘self-care’ (85.7 from 6 RCTs) and ‘CBT’ (83.8 from 19 RCTs) were relatively higher (Fig. 3C; Table 2). Given the ongoing debate about which treatment methods should be used for managing ME/CFS [48], this finding might stem from that non-pharmacological interventions can be utilized for managing ME/CFS patients, particularly those experiencing severe levels of fatigue, as recommended by NICE [49]. Unlike age (R^2^ = 0.003, p = 0.681), the type of intervention (R^2^ = 0.327, p = 0.011) demonstrated significant influence on fatigue severity scores in ME/CFS patients according to the linear mixed-effects model (see Additional file 7: Table S4).

Although ME/CFS has been defined as a complex neurological disease, concerns about its heterogeneity have led to the use of various case definitions. Out of the 25 currently used case definitions, we observed the application of four different case definitions in our study, including the 1994 CDC criteria (55 RCTs). Among these four case definitions, the Oxford criteria are the most lenient, primarily focusing on fatigue-related symptoms such as sleep disturbance, while the Canadian criteria encompass a broader range of pathological symptoms including anorexia, cardiovascular symptoms, and gastrointestinal symptoms [50]. The 1994 CDC criteria are generally considered moderately stricter, diagnosed by the presence of 4 or more symptoms encompassing the essential fatigue-related symptoms as well as four regional pains [50] Interestingly, participants in 2 RCTs using the Canadian criteria showed the highest average fatigue score of 83.6 (Fig. 4A; Table 2). Fatigue severities also notably differed according to each assessment tool; with relatively severe levels of fatigue observed in patients of RCTs using CIS (88.6 from 20 RCTs), while the lowest levels of fatigue were seen in patients of RCTs using MFI (68.8 from 9 RCTs) and one RCT that used MFS (54.2) (Fig. 4C; Table 2). CIS has been shown to effectively distinguish individuals with ME/CFS from those who do not suffer from ME/CFS based on the scores of each questionnaire’s criteria [51–53]. From our analyses using a linear mixed effect model, we found the notable influence between assessment tool (R^2^ = 0.437, p < 0.0001) and fatigue severity, but not by case definition (R^2^ = 0.084, p = 0.296) (Additional file 7: Table S4).

Based on the publication bias and quality assessment, we investigated the robustness of synthesized results. Then an additional meta-analysis after compensation of publication bias showed overall fatigue severity 71.5 (Additional file 5: Table S2), while fatigue severity was 77.2 after removing studies with high risk of bias (Additional file 6: Table S3). We herein produced a comprehensive feature of fatigue levels in patients with ME/CFS, but there are some limitations in our study. In order to obtain an objectively assessed fatigue data, we extracted only from RCTs. This means that we may have excluded the patients with extremely high or low levels of fatigue severity, as they may not have been able to easily participate in RCTs or may not have been suitable for assessing interventions. Thus, the data obtained from RCTs may not fully represent the real-world features of fatigue severity in ME/CFS patients. Another limitation is that different questionnaire-based assessment tools could reflect varying levels of fatigue severity due to the varying levels of sensitivity and specificity, even for the same fatigue score when converted into a maximum of 100. This strategy may affect the relatively high level of heterogeneity in our meta-analyzed data. Additionally, there may be a potential language bias as we excluded non-English studies due to concerns about our disability and quality issues. In future studies, it may be necessary to include longitudinal cohort studies to investigate changes in fatigue severity over time.

Despite the limitations above, our results firstly produced the overall features of fatigue severity in patients with ME/CFS. Our data will provide comparative insights not only for clinicians in the processes of diagnosis, therapeutic assessment and decision-making management of patients, but also for researchers involved in fatigue-related investigations.

### Supplementary Information

Supplementary Material 1.

Supplementary Material 2.

Supplementary Material 3.

Supplementary Material 4.

Supplementary Material 5.

Supplementary Material 6.

Supplementary Material 7.

Supplementary Material 8.

## Data Availability

All data related to this study are available in the public domain.
