# Correction: Systematic review of fatigue severity in ME/CFS patients: insights from randomized controlled trials

**DOI:** 10.1186/s12967-024-05390-6

**Published:** 2024-06-28

**Authors:** Jae‑Woong Park, Byung‑Jin Park, Jin‑Seok Lee, Eun‑Jung Lee, Yo‑Chan Ahn, Chang‑Gue Son

**Affiliations:** 1 Korean Medical College of Daejeon University, 62, Daehak‑Ro, Dong‑Gu, Daejeon, 34520 Republic of Korea; 2 Department of Korean Rehabilitation Medicine, College of Korean Medicine, Daejeon University, 176 Daedeok‑Daero, Seo‑Gu, Daejeon, 35235 Republic of Korea; 3 Department of Health Service Management, Daejeon University, Daejeon, Republic of Korea; 4 Research Center for CFS/ME, Daejeon Oriental Hospital of Daejeon University, 176 Daedeok‑Daero, Seo‑Gu, Daejeon, 35235 Republic of Korea; 5 Institute of Bioscience and Integrative Medicine, Daejeon University, 62 Daehak‑Ro, Dong‑Gu, Daejeon, 34520 Republic of Korea

**Correction** **:** **Journal of Translational Medicine (2024) 22:529** 10.1186/s12967-024-05349-7

Following publication of the original article [1], we have been notified that there was a part of text highlighted in yellow by mistake in Fig. 1. It should not be yellow highlighted.

It is now as follows:

**Fig. 1:**
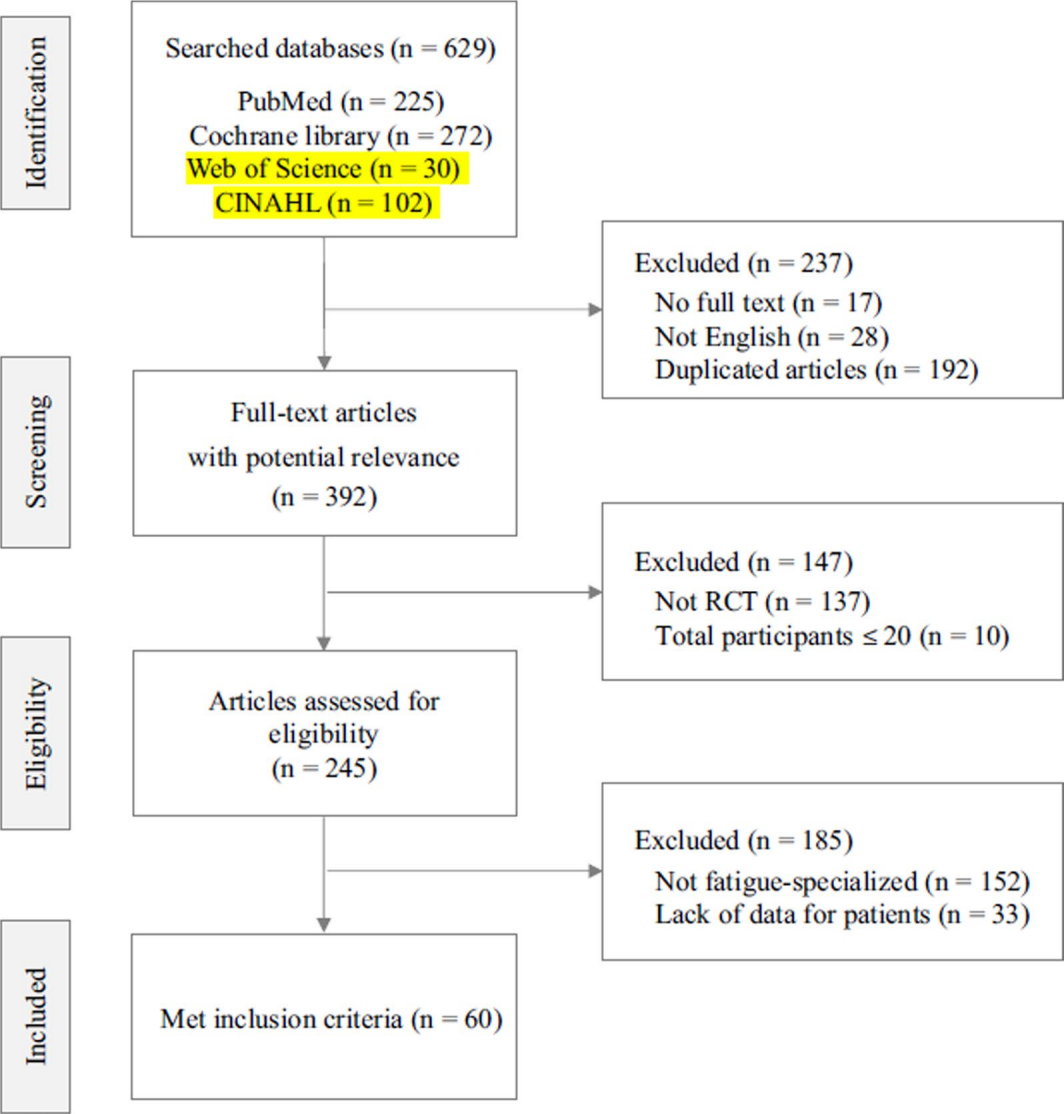
Flow chart of study selection diagram. n: Number of study

The original article was updated.
