# Supplementary material for: Systematic review of fatigue severity in ME/CFS patients: insights from randomized controlled trials
Source: J Transl Med. 2024 Jun 3;22:529. doi: 10.1186/s12967-024-05349-7 (PMC11145935; doi:10.1186/s12967-024-05349-7)
Supplement: Supplementary file 1 — Supplementary Material 1. [file 12967_2024_5349_MOESM1_ESM.pptx]

## Slide 1
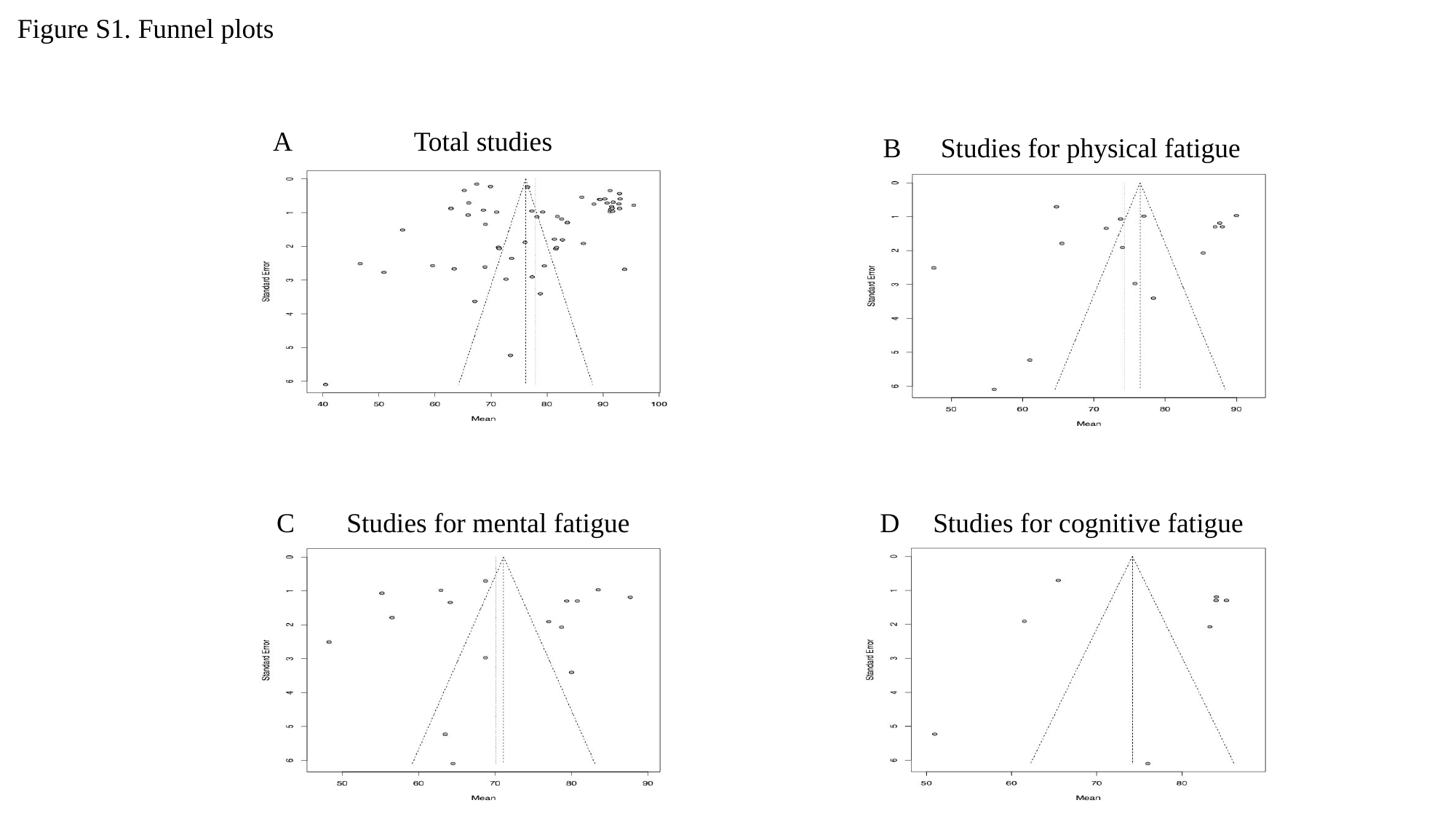

Figure S1. Funnel plots
A
Total studies
B
Studies for physical fatigue
C
Studies for mental fatigue
D
Studies for cognitive fatigue
