# Supplementary figures and images for: Systematic review of fatigue severity in ME/CFS patients: insights from randomized controlled trials
Source: J Transl Med. 2024 Jun 3;22:529. doi: 10.1186/s12967-024-05349-7 (PMC11145935; doi:10.1186/s12967-024-05349-7)

## Slide 1
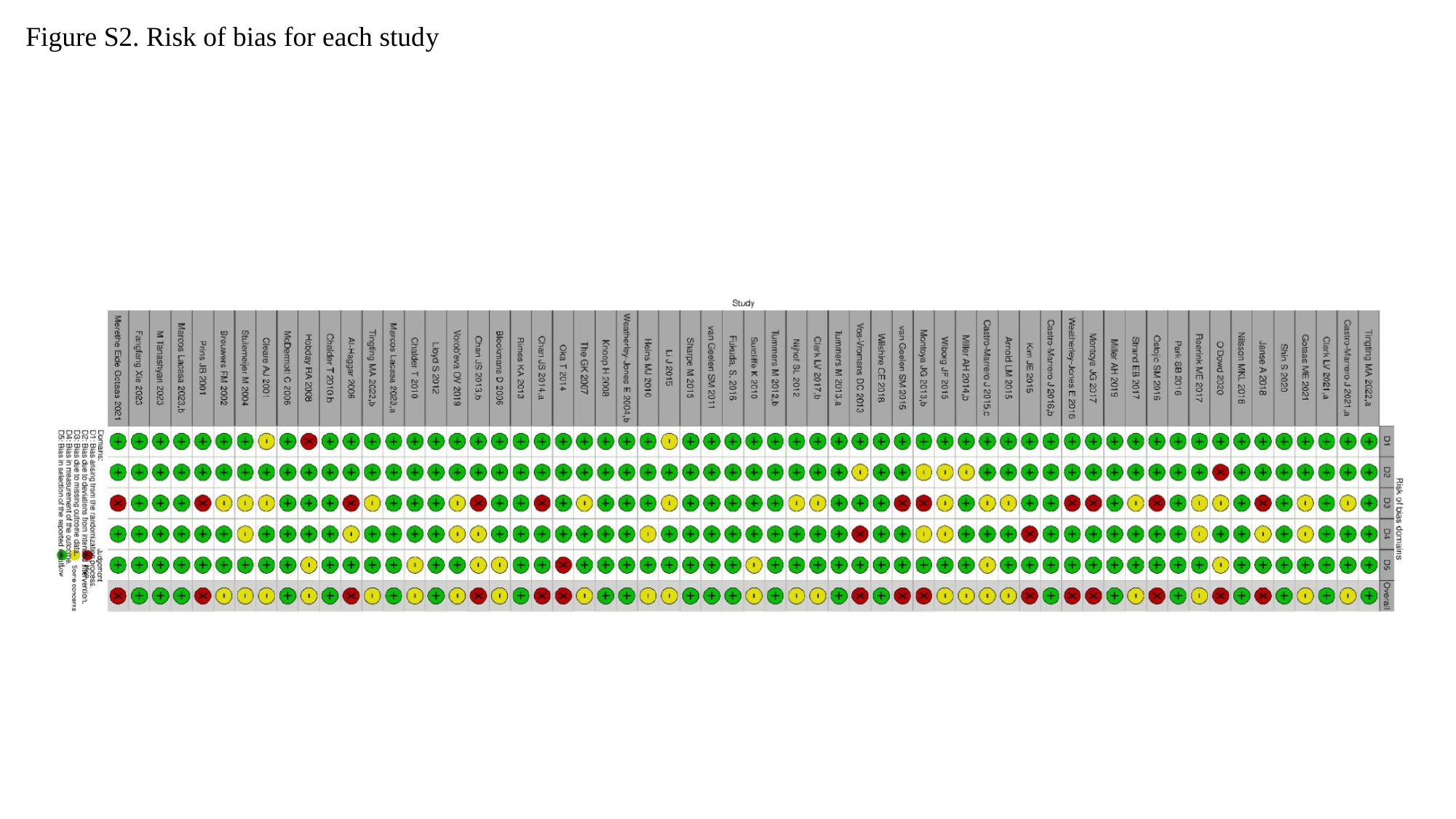

Figure S2. Risk of bias for each study

Supplement: Supplementary file 2 — Supplementary Material 2. [file 12967_2024_5349_MOESM2_ESM.pptx]
