# Supplementary material for: Systematic review of fatigue severity in ME/CFS patients: insights from randomized controlled trials
Source: J Transl Med. 2024 Jun 3;22:529. doi: 10.1186/s12967-024-05349-7 (PMC11145935; doi:10.1186/s12967-024-05349-7)
Supplement: Supplementary file 3 — Supplementary Material 3. [file 12967_2024_5349_MOESM3_ESM.pptx]

## Slide 1
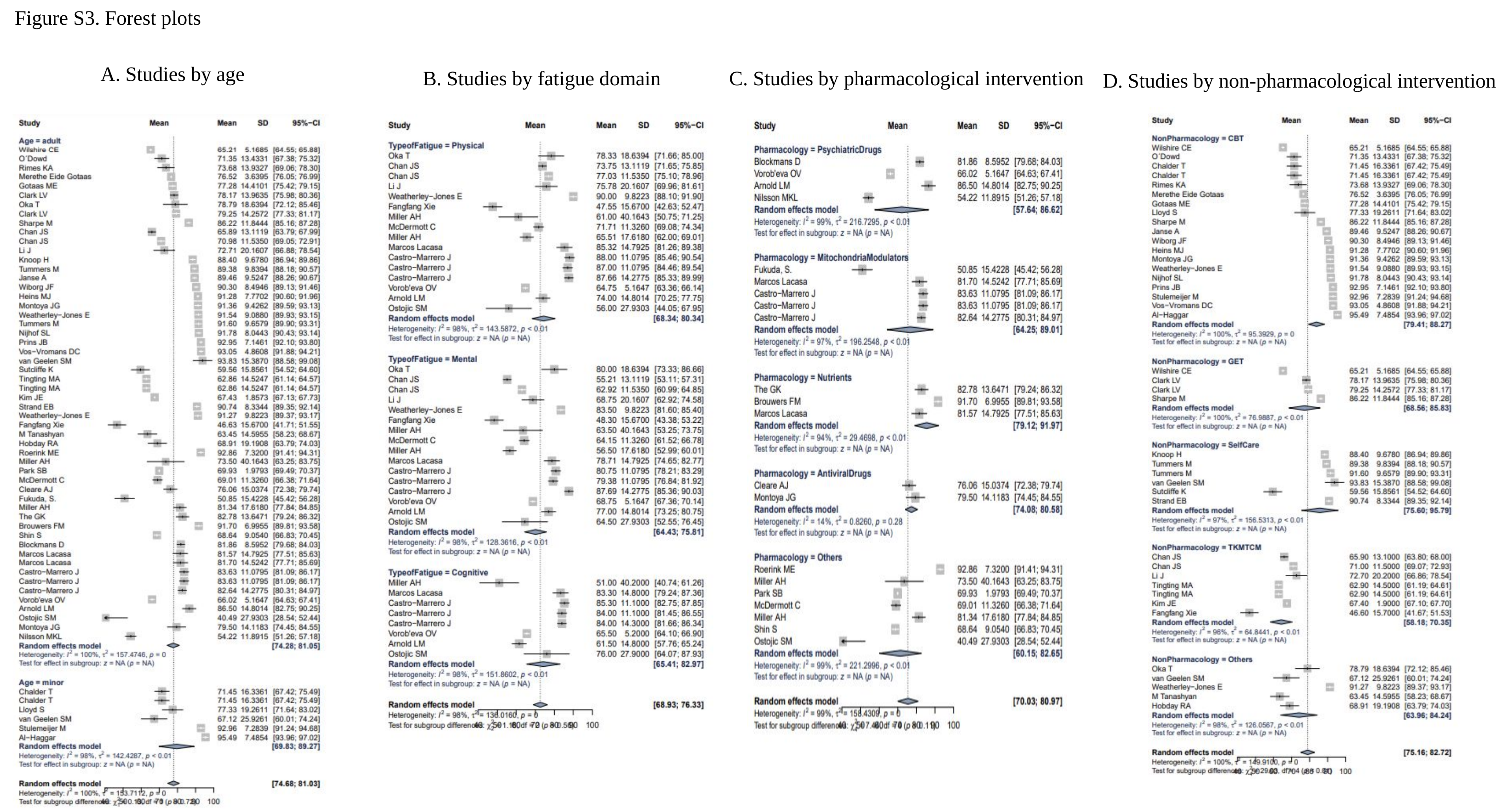

Figure S3. Forest plots
A. Studies by age
B. Studies by fatigue domain
C. Studies by pharmacological intervention
D. Studies by non-pharmacological intervention
