# Supplementary material for: Systematic review of fatigue severity in ME/CFS patients: insights from randomized controlled trials
Source: J Transl Med. 2024 Jun 3;22:529. doi: 10.1186/s12967-024-05349-7 (PMC11145935; doi:10.1186/s12967-024-05349-7)
Supplement: Supplementary file 4 — Supplementary Material 4. [file 12967_2024_5349_MOESM4_ESM.docx]

| **Table S1. Fatigue score by assessment tools used in RCTs** | | | | | |
| --- | --- | --- | --- | --- | --- |
| Assessment tool  (N.of studies) | RCTs | Case definition | Type of intervention | Raw score | Converted score  (out of 100) |
|  |  |  |  | Mean ± SD | Mean ± SD |
| CFQ  (20) | Wilshire CE [1] | 1994 CDC, Oxford | CBT/GET | 21.5±1.7 | 65.2±5.1 |
|  | Hobday RA [2] | 1994 CDC | Low sugar diet | 22.7±6.3 | 68.9±19.1 |
|  | McDermott C [3] | 1994 CDC | Immunomodulator | 22.7±3.7 | 69±11.3 |
|  | O’Dowd [4] | Not defined | CBT & psycho-education | 23.5±4.4 | 71.3±13.4 |
|  | Chalder T [5] | 1994 CDC, Oxford | CBT | 23.5±5.3 | 71.4±16.3 |
|  | Chalder T [6] | 1994 CDC, Oxford | CBT & psycho-education | 23.5±5.3 | 71.4±16.3 |
|  | Rimes KA [7] | 1994 CDC, Oxford | Mindfulness-based CBT | 24.3±4.5 | 73.6±13.9 |
|  | Cleare AJ [8] | 1994 CDC | Hydrocortisone | 25.1±4.9 | 76±15 |
|  | Merethe Eide Gotaas [9] | 1994 CDC, Canadian | CBT | 25.2±1.2 | 76.5±3.6 |
|  | Gotaas ME [10] | 1994 CDC | I-CBT | 25.5±4.7 | 77.2±14.4 |
|  | Lloyd S [11] | 1994 CDC, Oxford | CBT & psycho-education | 25.5±6.3 | 77.3±19.2 |
|  | Clark LV [12] | 1994 CDC | GET | 25.7±4.6 | 78.1±13.9 |
|  | Oka T [13] | 1994 CDC | Isometric yoga | 26±6.1 | 78.7±18.6 |
|  | Clark LV [14] | 1994 CDC, Oxford, | GET | 26.1±4.7 | 79.2±14.2 |
|  | Sharpe M [15] | Oxford | CBT/GET | 28.4±3.9 | 86.2±11.8 |
|  | Fukuda, S. [16] | 1994 CDC | Ubiquinol-10 (CoQ10) | 28.4±8.6^a^ | 50.8±15.4 |
|  | Chan JS [17] | 1994 CDC | Qigong | 36.9±7.3^a^ | 65.8±13.1 |
|  | Chan JS [18] | 1994 CDC | Qigong | 39.7±6.4^a^ | 70.9±11.5 |
|  | Li J [19] | 1994 CDC | Qigong | 40.7±11.2^a^ | 72.7±20.1 |
|  | Miller AH [20] | 1994 CDC | Myelophil | 45.5±9.8^a^ | 81.3±17.6 |
|  | Total |  |  | 24.6±0.5  38.2±2.9^a^ | 75±5.2 |
| CIS  (20) | van Geelen SM [21] | 1994 CDC | Self-confrontation method | 37.5±14.5 | 67.1±25.9 |
|  | The GK [22] | 1994 CDC | Acclydine | 46.3±7.6 | 82.7±13.6 |
|  | Knoop H [23] | 1994 CDC | Guided self-instruction | 49.5±5.4 | 88.3±9.6 |
|  | Tummers M [24] | 1994 CDC | Guided self-instruction | 50±5.5 | 89.3±9.8 |
|  | Janse A [25] | 1994 CDC | CBT | 50.1±5.3 | 89.4±9.5 |
|  | Wiborg JF [26] | 1994 CDC | CBT | 50.5±4.7 | 90.2±8.4 |
|  | Heins MJ [27] | 1994 CDC | CBT | 51.1±4.3 | 91.2±7.7 |
|  | Montoya JG [28] | 1994 CDC | MRT/CBT | 51.1±5.2 | 91.3±9.4 |
|  | Weatherley-Jones E [29] | 1994 CDC | MRT/CBT | 51.2±5 | 91.5±9 |
|  | Tummers M [30] | 1994 CDC | Guided self-instruction | 51.2±5.4 | 91.6±9.6 |
|  | Brouwers FM [31] | 1994 CDC | Polynutrient supplement | 51.3±3.9 | 91.6±6.9 |
|  | Nijhof SL [32] | 1994 CDC | FITNET | 51.3±4.5 | 91.7±8 |
|  | Roerink ME [33] | 1994 CDC | IL-12p40 / CSF-1 | 52±4 | 92.8±7.3 |
|  | Prins JB [34] | 1994 CDC | CBT | 52±4 | 92.9±7.1 |
|  | Stulemeijer M [35] | 1994 CDC | CBT | 52±4 | 92.9±7.2 |
|  | Vos-Vromans DC [36] | 1994 CDC | CBT | 52.1±2.7 | 93±4.8 |
|  | van Geelen SM [37] | 1994 CDC | Activity Pacing Self-Management | 52.5±8.6 | 93.8±15.3 |
|  | Al-Haggar [38] | 1994 CDC | CBT | 53.4±4.2 | 95.4±7.4 |
|  | Shin S [39] | 1994 CDC | SJDBT | 96.1±12.7^b^ | 68.6±9 |
|  | Blockmans D [40] | 1994 CDC | Methylphenidate | 114.6±12^b^ | 81.8±8.5 |
|  | Total |  |  | 50.6±1.1  105.3±9.2^b^ | 72.7±7.9 |
| FIS  (6) | Sutcliffe K [41] | 1994 CDC | Home-orthostatic training | 95.3±25.3 | 59.5±15.8 |
|  | Castro-Marrero J [42] | 1994 CDC | Melatonin with zinc supplement | 130.5±23.6 | 81.6±14.8 |
|  | Marcos Lacasa [43] | 1994 CDC | Yeast -Beta glucan with vitamins | 130.7±23.2 | 81.7±14.5 |
|  | Castro-Marrero J [44] | 1994 CDC | oral coenzyme Q10 + NADH | 133.8±17.7 | 83.6±11 |
|  | Castro-Marrero J [45] | 1994 CDC | oral coenzyme Q10 + NADH | 133.8±17.7 | 83.6±11 |
|  | Castro-Marrero J [46] | 1994 CDC | oral coenzyme Q10 + NADH | 132.2±22.8 | 82.6±14.2 |
|  | Total |  |  | 125.9±16.2 | 78.7±10.2 |
| FSS  (5) | Tingting MA [47] | 1994 CDC | Herb, Moxibustion, Acupuncture | 39.6±9.1 | 62.8±14.5 |
|  | Tingting MA [48] | 1994 CDC | Moxibustion | 39.6±9.1 | 62.8±14.5 |
|  | Kim JE [49] | 1994 CDC | Acupuncture | 42.4±1.1 | 67.4±1.8 |
|  | Park SB [50] | 1994 CDC | Human placental extract | 44±1.2 | 69.9±1.9 |
|  | Strand EB [51] | CDC, Canadian | Group-based self management | 57.1±5.2 | 90.7±8.3 |
|  | Total |  |  | 44.5±3.2 | 70.7±11.6 |
| MFI  (8) | Vorob'eva OV [52] | Not defined | Noofen | 66±5.1 | 66±5.1 |
|  | Miller AH [53] | 1994 CDC | Antiinflammatory drug | 14.7±8^c^ | 73.5±40.1 |
|  | Arnold LM [54] | 1994 CDC | Duloxetine | 17.3±2.9^c^ | 86.5±14.8 |
|  | Weatherley-Jones E [55] | Oxford | Homeopathic medicine | 18.2±1.9^c^ | 91.2±9.8 |
|  | Ostojic SM [56] | 1994 CDC | GAA supplement | 40.4±27.9 | 40.4±27.9 |
|  | Fangfang Xie [57] | 1994 CDC | Yijinjing Qigong | 46.6±15.6 | 46.6±15.6 |
|  | M Tanashyan [58] | 1994 CDC | Neuroprotective therapy | 63.4±14.5 | 63.4±14.5 |
|  | Montoya JG [58, 59] | 1994 CDC | Valganciclovir | 79.5±14.1 | 79.5±14.1 |
|  | Total |  |  | 59.7±6.9  16.9±0.9^c^ | 70±9.7 |
| MFS  (1) | Nilsson MKL [60] |  | Monoaminergic stabilizer | 24.4±5.4 | 54.2±11.9 |
|  | Total |  |  | 24.4±5.4 | 54.2±11.9 |
| ^a^ This CFQ is a modified version. (This survey consists of 14 questions with a rating scale of 0 to 4points for each item. (total score range  0–56)  ^b^ This study used modified version of CIS, which is ranking from 20 to 140.  ^c^  This study used modified version of MFI, which is ranking from 0 to 20. | | | | | |

**Supplemental References**

1. Wilshire, C.E., et al., *Rethinking the treatment of chronic fatigue syndrome—a reanalysis and evaluation of findings from a recent major trial of graded exercise and CBT.* BMC psychology, 2018. **6**(1): p. 1-12.

2. Hobday, R., et al., *Dietary intervention in chronic fatigue syndrome.* Journal of human nutrition and dietetics, 2008. **21**(2): p. 141-149.

3. McDermott, C., et al., *A placebo-controlled, double-blind, randomized controlled trial of a natural killer cell stimulant (BioBran MGN-3) in chronic fatigue syndrome.* Journal of the Association of Physicians, 2006. **99**(7): p. 461-468.

4. O’Dowd, H., et al., *The feasibility and acceptability of an early intervention in primary care to prevent chronic fatigue syndrome (CFS) in adults: randomised controlled trial.* Pilot and Feasibility Studies, 2020. **6**(1): p. 1-12.

5. Burgess, M., M. Andiappan, and T. Chalder, *Cognitive behaviour therapy for chronic fatigue syndrome in adults: Face to face versus telephone treatment-A randomized controlled trial.* Behavioural and cognitive psychotherapy, 2012. **40**(2): p. 175-191.

6. Chalder, T., et al., *Family-focused cognitive behaviour therapy versus psycho-education for chronic fatigue syndrome in 11-to 18-year-olds: a randomized controlled treatment trial.* Psychological medicine, 2010. **40**(8): p. 1269-1279.

7. Rimes, K.A. and J. Wingrove, *Mindfulness‐based cognitive therapy for people with chronic fatigue syndrome still experiencing excessive fatigue after cognitive behaviour therapy: a pilot randomized study.* Clinical psychology & psychotherapy, 2013. **20**(2): p. 107-117.

8. Cleare, A., V. O'Keane, and J. Miell, *Plasma leptin in chronic fatigue syndrome and a placebo‐controlled study of the effects of low‐dose hydrocortisone on leptin secretion.* Clinical endocrinology, 2001. **55**(1): p. 113-119.

9. Gotaas, M.E., et al., *Cognitive behavioral therapy improves physical function and fatigue in mild and moderate chronic fatigue syndrome: a consecutive randomized controlled trial of standard and short interventions.* Frontiers in psychiatry, 2021. **12**: p. 580924.

10. Gotaas, M.E., et al., *Characteristics associated with physical functioning and fatigue in patients with chronic fatigue syndrome (CFS): secondary analyses of a randomized controlled trial.* Fatigue: Biomedicine, Health & Behavior, 2023: p. 1-17.

11. Lloyd, S., T. Chalder, and K.A. Rimes, *Family-focused cognitive behaviour therapy versus psycho-education for adolescents with chronic fatigue syndrome: long-term follow-up of an RCT.* Behaviour research and therapy, 2012. **50**(11): p. 719-725.

12. Clark, L.V., et al., *Guided graded exercise self-help for chronic fatigue syndrome: Long term follow up and cost-effectiveness following the GETSET trial.* Journal of Psychosomatic Research, 2021. **146**: p. 110484.

13. Oka, T., et al., *Isometric yoga improves the fatigue and pain of patients with chronic fatigue syndrome who are resistant to conventional therapy: a randomized, controlled trial.* BioPsychoSocial medicine, 2014. **8**(1): p. 1-9.

14. Clark, L.V., et al., *Guided graded exercise self-help plus specialist medical care versus specialist medical care alone for chronic fatigue syndrome (GETSET): a pragmatic randomised controlled trial.* The Lancet, 2017. **390**(10092): p. 363-373.

15. Sharpe, M., et al., *Rehabilitative treatments for chronic fatigue syndrome: long-term follow-up from the PACE trial.* The Lancet Psychiatry, 2015. **2**(12): p. 1067-1074.

16. Fukuda, S., et al., *Ubiquinol‐10 supplementation improves autonomic nervous function and cognitive function in chronic fatigue syndrome.* Biofactors, 2016. **42**(4): p. 431-440.

17. Chan, J.S., et al., *Qigong exercise alleviates fatigue, anxiety, and depressive symptoms, improves sleep quality, and shortens sleep latency in persons with chronic fatigue syndrome-like illness.* Evidence-Based Complementary and Alternative Medicine, 2014. **2014**.

18. Chan, J.S., et al., *Effects of qigong exercise on fatigue, anxiety, and depressive symptoms of patients with chronic fatigue syndrome-like illness: a randomized controlled trial.* Evidence-Based Complementary and Alternative Medicine, 2013. **2013**.

19. Li, J., et al., *From body to mind and spirit: Qigong exercise for bereaved persons with chronic fatigue syndrome-like illness.* Evidence-Based Complementary and Alternative Medicine, 2015. **2015**.

20. Joung, J.-Y., et al., *The efficacy and safety of myelophil, an ethanol extract mixture of astragali radix and salviae radix, for chronic fatigue syndrome: a randomized clinical trial.* Frontiers in Pharmacology, 2019. **10**: p. 991.

21. van Geelen, S.M., et al., *Self-investigation in adolescent chronic fatigue syndrome: Narrative changes and health improvement.* Patient education and counseling, 2011. **83**(2): p. 227-233.

22. The, G.K.H., G. Bleijenberg, and J.W.M. van der Meer, *The effect of acclydine in chronic fatigue syndrome: a randomized controlled trial.* PLoS Clinical Trials, 2007. **2**(5): p. e19.

23. Knoop, H., J.W. van der Meer, and G. Bleijenberg, *Guided self-instructions for people with chronic fatigue syndrome: randomised controlled trial.* The British Journal of Psychiatry, 2008. **193**(4): p. 340-341.

24. Tummers, M., et al., *Moderators of the treatment response to guided self-instruction for chronic fatigue syndrome.* Journal of Psychosomatic Research, 2013. **74**(5): p. 373-377.

25. Janse, A., et al., *Efficacy of web-based cognitive–behavioural therapy for chronic fatigue syndrome: randomised controlled trial.* The British Journal of Psychiatry, 2018. **212**(2): p. 112-118.

26. Wiborg, J.F., et al., *Randomised controlled trial of cognitive behaviour therapy delivered in groups of patients with chronic fatigue syndrome.* Psychotherapy and psychosomatics, 2015. **84**(6): p. 368-376.

27. Heins, M.J., et al., *Possible detrimental effects of cognitive behaviour therapy for chronic fatigue syndrome.* Psychotherapy and Psychosomatics, 2010. **79**(4): p. 249-256.

28. Vos-Vromans, D., et al., *Economic evaluation of multidisciplinary rehabilitation treatment versus cognitive behavioural therapy for patients with chronic fatigue syndrome: A randomized controlled trial.* PLoS One, 2017. **12**(6): p. e0177260.

29. Vos‐Vromans, D., et al., *Multidisciplinary rehabilitation treatment versus cognitive behavioural therapy for patients with chronic fatigue syndrome: a randomized controlled trial.* Journal of internal medicine, 2016. **279**(3): p. 268-282.

30. Tummers, M., et al., *Implementing a minimal intervention for chronic fatigue syndrome in a mental health centre: a randomized controlled trial.* Psychological Medicine, 2012. **42**(10): p. 2205-2215.

31. Brouwers, F., et al., *The effect of a polynutrient supplement on fatigue and physical activity of patients with chronic fatigue syndrome: a double‐blind randomized controlled trial.* Qjm, 2002. **95**(10): p. 677-683.

32. Nijhof, S.L., et al., *Effectiveness of internet-based cognitive behavioural treatment for adolescents with chronic fatigue syndrome (FITNET): a randomised controlled trial.* The Lancet, 2012. **379**(9824): p. 1412-1418.

33. Roerink, M.E., et al., *Cytokine signatures in chronic fatigue syndrome patients: a case control study and the effect of anakinra treatment.* Journal of translational medicine, 2017. **15**: p. 1-10.

34. Prins, J.B., et al., *Cognitive behaviour therapy for chronic fatigue syndrome: a multicentre randomised controlled trial.* The Lancet, 2001. **357**(9259): p. 841-847.

35. Stulemeijer, M., et al., *Cognitive behaviour therapy for adolescents with chronic fatigue syndrome: randomised controlled trial.* Bmj, 2004. **330**(7481): p. 14.

36. Vos-Vromans, D.C., et al., *Differences in physical functioning between relatively active and passive patients with Chronic Fatigue Syndrome.* Journal of Psychosomatic Research, 2013. **75**(3): p. 249-254.

37. Kos, D., et al., *Activity pacing self-management in chronic fatigue syndrome: a randomized controlled trial.* The American Journal of Occupational Therapy, 2015. **69**(5): p. 6905290020p1-6905290020p11.

38. Al-Haggar, M.S., Z.A. Al-Naggar, and M.A. Abdel-Salam, *Biofeedback and cognitive behavioral therapy for Egyptian adolescents suffering from chronic fatigue syndrome.* Journal of pediatric neurology, 2006. **4**(03): p. 161-169.

39. Shin, S., S.J. Park, and M. Hwang, *Effectiveness a herbal medicine (Sipjeondaebo-tang) on adults with chronic fatigue syndrome: A randomized, double-blind, placebo-controlled trial.* Integrative Medicine Research, 2021. **10**(2): p. 100664.

40. Blockmans, D., et al., *Does methylphenidate reduce the symptoms of chronic fatigue syndrome?* The American journal of medicine, 2006. **119**(2): p. 167. e23-167. e30.

41. Sutcliffe, K., et al., *Home orthostatic training in chronic fatigue syndrome–a randomized, placebo‐controlled feasibility study.* European journal of clinical investigation, 2010. **40**(1): p. 18-24.

42. Castro-Marrero, J., et al., *Effect of melatonin plus zinc supplementation on fatigue perception in myalgic encephalomyelitis/chronic fatigue syndrome: a randomized, double-blind, placebo-controlled trial.* Antioxidants, 2021. **10**(7): p. 1010.

43. Lacasa, M., et al., *Yeast Beta-Glucan Supplementation with Multivitamins Attenuates Cognitive Impairments in Individuals with Myalgic Encephalomyelitis/Chronic Fatigue Syndrome: A Randomized, Double-Blind, Placebo-Controlled Trial.* Nutrients, 2023. **15**(21): p. 4504.

44. Castro-Marrero, J., et al., *Effect of dietary coenzyme Q10 plus NADH supplementation on fatigue perception and health-related quality of life in individuals with myalgic encephalomyelitis/chronic fatigue syndrome: a prospective, randomized, double-blind, placebo-controlled trial.* Nutrients, 2021. **13**(8): p. 2658.

45. Castro-Marrero, J., et al., *Effect of coenzyme Q10 plus nicotinamide adenine dinucleotide supplementation on maximum heart rate after exercise testing in chronic fatigue syndrome–A randomized, controlled, double-blind trial.* Clinical Nutrition, 2016. **35**(4): p. 826-834.

46. Castro-Marrero, J., et al., *Does oral coenzyme Q10 plus NADH supplementation improve fatigue and biochemical parameters in chronic fatigue syndrome?* 2015, Mary Ann Liebert, Inc. 140 Huguenot Street, 3rd Floor New Rochelle, NY 10801 USA.

47. Tingting, M., et al., *Ginger-indirect moxibustion plus acupuncture versus acupuncture alone for chronic fatigue syndrome: a randomized controlled trial.* Journal of Traditional Chinese Medicine, 2022. **42**(2): p. 242.

48. Ma, T., et al., *Dose-Effect of Long-Snake-Like Moxibustion for Chronic Fatigue Syndrome: Study Protocol for a Randomized Controlled Trial.* 2021.

49. Kim, J.-E., et al., *Acupuncture for chronic fatigue syndrome and idiopathic chronic fatigue: a multicenter, nonblinded, randomized controlled trial.* Trials, 2015. **16**(1): p. 1-13.

50. Park, S.B., et al., *Human placental extract as a subcutaneous injection is effective in chronic fatigue syndrome: a multi-center, double-blind, randomized, placebo-controlled study.* Biological and Pharmaceutical Bulletin, 2016. **39**(5): p. 674-679.

51. Pinxsterhuis, I., et al., *Effectiveness of a group-based self-management program for people with chronic fatigue syndrome: a randomized controlled trial.* Clinical Rehabilitation, 2017. **31**(1): p. 93-103.

52. Vorob'eva, O. and V. Rusaya, *Efficacy and safety of noophen in the treatment of chronic fatigue syndrome in patients with cerebrovascular insufficiency.* Zhurnal Nevrologii i Psikhiatrii Imeni SS Korsakova, 2017. **117**(11): p. 31-36.

53. Miller, A.H., et al., *Decreased basal ganglia activation in subjects with chronic fatigue syndrome: association with symptoms of fatigue.* PLoS One, 2014. **9**(5): p. e98156.

54. Arnold, L.M., et al., *A randomized, placebo-controlled, double-blinded trial of duloxetine in the treatment of general fatigue in patients with chronic fatigue syndrome.* Psychosomatics, 2015. **56**(3): p. 242-253.

55. Weatherley-Jones, E., et al., *A randomised, controlled, triple-blind trial of the efficacy of homeopathic treatment for chronic fatigue syndrome.* Journal of psychosomatic research, 2004. **56**(2): p. 189-197.

56. Ostojic, S.M., et al., *Supplementation with guanidinoacetic acid in women with chronic fatigue syndrome.* Nutrients, 2016. **8**(2): p. 72.

57. Xie, F., et al., *Effects of Yijinjing Qigongin Alleviating Fatigue, Sleep Quality, and Health Status on Patients with Chronic Fatigue Syndrome: A Randomized, Controlled, and Parallel Group Clinical Study.* Complementary Medicine Research, 2023: p. 1-9.

58. Tanashyan, M., et al., *A prospective randomized, double-blind placebo-controlled study to evaluate the effectiveness of neuroprotective therapy using functional brain MRI in patients with post-covid chronic fatigue syndrome.* Biomedicine & Pharmacotherapy, 2023. **168**: p. 115723.

59. Montoya, J.G., et al., *Randomized clinical trial to evaluate the efficacy and safety of valganciclovir in a subset of patients with chronic fatigue syndrome.* Journal of medical virology, 2013. **85**(12): p. 2101-2109.

60. Nilsson, M.K.L., et al., *A randomised controlled trial of the monoaminergic stabiliser (−)-OSU6162 in treatment of myalgic encephalomyelitis/chronic fatigue syndrome.* Acta Neuropsychiatrica, 2018. **30**(3): p. 148-157.
