# Supplementary material for: Systematic review of fatigue severity in ME/CFS patients: insights from randomized controlled trials
Source: J Transl Med. 2024 Jun 3;22:529. doi: 10.1186/s12967-024-05349-7 (PMC11145935; doi:10.1186/s12967-024-05349-7)
Supplement: Supplementary file 5 — Supplementary Material 5. [file 12967_2024_5349_MOESM5_ESM.docx]

| Table S2. Results of Egger’s test | | | |  |
| --- | --- | --- | --- | --- |
| Item | Egger’s test | | Triam and fill |  |
|  | Regression intercept [standard error] | P score | Adjusted Score |  |
| Total study | 7.1 [3.1] | 0.0242 | 71.5 [67.9; 75.1] |  |
| Age |  |  |  |  |
| Adults (40±5.7) | 7.4 [3.3] | 0.0308 | 71.5 [67.7; 75.4] |  |
| Minors (15±0.9) | -12.5 [2.6] | 0.0083 | 92.0 [78.1; 105.9] |  |
| Type of Fatigue |  |  |  |  |
| Physical | -0.7 [4.5] | 0.8734 | 77.2 [70.5; 84.0] |  |
| Mental | -0.7 [4.5] | 0.8800 | 70.1 [64.4; 75.8] |  |
| Cognitive | | 2.8 [5.8] | 0.6424 | 74.2 [65.4; 83.0] |
| Type of Intervention |  |  |  |  |
| Pharmacology | 4.7 [3.0] | 0.1383 | 71.1 [65.4; 76.8] |  |
| Psychiatric drugs | 7.6 [17.0] | 0.6986 | 67.8 [53.7; 81.9] |  |
| Mitochondria modulators | -15.3 [5.8] | 0.0795 | 76.6 [64.3; 89.0] |  |
| Nutrients | -9.7 [0.8] | 0.0555 | 91.7 [83.4; 100.0] |  |
| Antiviral drugs |  |  | 71.4 [60.2; 82.7] |  |
| Others | 4.0 [6.7] | 0.5759 | 74.7 [67.4; 82.0] |  |
| Non- Pharmacology | 9.6 [4.4] | 0.0378 | 75.2 [71.1; 79.2] |  |
| CBT | 8.6 [7.7] | 0.2771 | 83.1 [78.7; 87.5] |  |
| GET | 23.6 [22.0] | 0.3961 | 68.1 [55.4; 80.8] |  |
| Self-care | -6.2 [5.0] | 0.2796 | 85.7 [75.6; 95.8] |  |
| TKM/TCM | -2.8 [2.2] | 0.2723 | 67.1 [59.3; 75.0] |  |
| Others | -10.2 [2.9] | 0.0384 | 89.0 [73.4; 104.6] |  |
| Case definition |  |  |  |  |
| 1994 CDC | 7.2 [3.1] | 0.0264 | 73.8 [70.3; 77.3] |  |
| Oxford | 8.1 [8.5] | 0.3725 | 66.4 [57.7; 75.1] |  |
| ICC | NA | NA | NA |  |
| Canadain criteria | NA | NA | NA |  |
| Not defined | NA | NA | NA |  |
| Assessment tool |  |  |  |  |
| CFQ | -0.5 [2.9] | 0.8667 | 74.3 [70.5; 78.1] |  |
| CIS | -5.6 [3.4] | 0.1175 | 88.6 [85.4; 91.8] |  |
| FSS | 8.5 [13.7] | 0.5809 | 70.8 [60.6; 80.9] |  |
| FIS | -9.8 [4.6] | 0.0994 | 78.9 [71.6; 86.3] |  |
| MFI | -1.9 [6.2] | 0.7703 | 72.8 [59.1; 86.5] |  |
| MFS | NA | NA | NA |  |
| Continent |  |  |  |  |
| Europe | 0.5 [3.4] | 0.8772 | 80.3 [76.9; 83.7] |  |
| Asia | -1.1 [2.2] | 0.6103 | 68.3 [62.2; 74.5] |  |
| North America | NA | NA | NA |  |
| Africa | NA | NA | NA |  |
| Year |  |  |  |  |
| Before 2010 | -11.8 [3.6] | 0.0094 | 91.5 [84.2; 98.8] |  |
| Since 2010 | 6.3 [3.3] | 0.0617 | 73.5 [69.8; 77.1] |  |
