# Supplementary material for: Systematic review of fatigue severity in ME/CFS patients: insights from randomized controlled trials
Source: J Transl Med. 2024 Jun 3;22:529. doi: 10.1186/s12967-024-05349-7 (PMC11145935; doi:10.1186/s12967-024-05349-7)
Supplement: Supplementary file 6 — Supplementary Material 6. [file 12967_2024_5349_MOESM6_ESM.docx]

| Table S3. Sensitivity analysis, removing RCTs with high risk of bias | | |  |
| --- | --- | --- | --- |
| Item | Fatigue score | |  |
|  | Mean [95% CI] | I^2^ |  |
| Total study | 77.2 [73.6; 80.77] | 99.5% |  |
| Age |  |  |  |
| Adults (40±5.7) | 77.3 [73.4; 81.2] | 99.6% |  |
| Minors (15±0.9) | 76.3 [67.2; 85.4] | 98.0% |  |
| Type of Fatigue |  |  |  |
| Physical | 75.1 [67.5; 82.6] | 98.8% |  |
| Mental | 71.6 [64.9; 78.3] | 98.2% |  |
| Cognitive | | 73.9 [64.0; 83.9] | 98.6% |
| Type of Intervention |  |  |  |
| Pharmacology | 75.5 [70.0; 81.0] | 98.6% |  |
| Psychiatric drugs | 72.1 [57.6; 86.6] | 99.1% |  |
| Mitochondria modulators | 76.6 [64.3; 89.0] | 96.9% |  |
| Nutrients | 85.5 [79.1; 92.0] | 93.8% |  |
| Antiviral drugs | 76.1 [72.4; 79.7] | NA |  |
| Others | 76.0 [68.0; 83.9] | 99.5% |  |
| Non- Pharmacology | 77.5 [72.4; 82.5] | 99.6% |  |
| CBT | 80.9 [75.0; 86.9] | 99.7% |  |
| GET | 77.2 [68.6; 85.8] | 99.7% |  |
| Self-care | 84.1 [72.3; 95.9] | 97.3% |  |
| TKM/TCM | 61.3 [50.9; 71.6] | 94.2% |  |
| Others | 74.2 [56.8; 91.5] | 98.4% |  |
| Case definition |  |  |  |
| 1994 CDC | 76.9 [73.2; 80.6] | 99.5% |  |
| Oxford | 77.1 [71.0; 83.1] | 99.5% |  |
| ICC | 54.2 [51.3; 57.2] | NA |  |
| Canadain diagostic criteria | 90.7 [89.3; 92.1] | NA |  |
| Not defined | 66.0 [64.6; 67.4] | NA |  |
| Assessment tool |  |  |  |
| CFQ | 73.7 [69.4; 78.0] | 99.0% |  |
| CIS | 86.9 [82.6; 91.3] | 98.1% |  |
| FSS | 71.6 [58.7; 84.5] | 99.7% |  |
| FIS | 78.9 [71.6; 86.3] | 93.7% |  |
| MFI | 71.3 [58.1; 84.5] | 99.2% |  |
| MFS | 54.2 [51.3; 57. 2] | 0 |  |
| Continent |  |  |  |
| Europe | 79.9 [76.4; 83.4] | 99.4% |  |
| Asia | 64.6 [56.8; 72.3] | 97.6% |  |
| North America | 86.5 [82. 8; 90.2] | 79.0% |  |
| Africa |  | 0 |  |
| Year |  |  |  |
| Before 2010 | 84.3 [78.4; 90.2] | 97.9% |  |
| Since 2010 | 75.6 [71.6; 79.6] | 99.6% |  |
