# Supplementary material for: Systematic review of fatigue severity in ME/CFS patients: insights from randomized controlled trials
Source: J Transl Med. 2024 Jun 3;22:529. doi: 10.1186/s12967-024-05349-7 (PMC11145935; doi:10.1186/s12967-024-05349-7)
Supplement: Supplementary file 7 — Supplementary Material 7. [file 12967_2024_5349_MOESM7_ESM.docx]

| Table S4. Linear-mixed effect model across fatigue and other factors | | | | | | | | |
| --- | --- | --- | --- | --- | --- | --- | --- | --- |
| Item | Total Fatigue | | Physical Fatigue | | Mental Fatigue | | Cognitive Fatigue | |
|  | R^2^ | p-value | R^2^ | p-value | R^2^ | p-value | R^2^ | p-value |
| Physical Fatigue | 0.4742 | 0.5033 | - | - | - | - | - | - |
| Mental Fatigue | 0.6264 | 0.0143 | 0.6731 | < 0.0001 | - | - | - | - |
| Cognitive Fatigue | 0.5421 | < 0.0001 | 0.5033 | 0.0425 | 0.5007 | 0.0544 | - | - |
| Age | 0.0033 | 0.6812 | 0.1373 | 0.1576 | 0.1839 | 0.0975 | 0.0361 | 0.6524 |
| Continent | 0.2162 | 0.0032 | 0.0799 | 0.5820 | 0.3195 | 0.0819 | 0.1477 | 0.3472 |
| Intervention | 0.3277 | 0.0011 | 0.0222 | 0.9917 | 0.5529 | 0.0483 | 0.7264 | 0.1268 |
| Assessment tool^a^ | 0.4371 | < 0.0001 | - | - | - | - | - | - |
| Case definition^b^ | 0.0840 | 0.2962 | - | - | - | - | - | - |

^a^ The correlation between the assessment tool and the fatigue domain could not be analyzed due to lack of information.

^b^ The correlation between the case definition and the fatigue domain could not be analyzed due to lack of information.
